# Effect of mobile text message reminders on routine childhood vaccination: a systematic review and meta-analysis

**DOI:** 10.1186/s13643-019-1054-0

**Published:** 2019-06-28

**Authors:** Zeleke Abebaw Mekonnen, Kassahun Alemu Gelaye, Martin C. Were, Kassahun Dessie Gashu, Binyam Chakilu Tilahun

**Affiliations:** 1 Department of Health Informatics, Institute of Public Health, College of Medicine and Health Sciences, University of Gondar, Gondar, Ethiopia; 2 Department of Epidemiology and Biostatistics, Institute of Public Health, College of Medicine and Health Sciences, University of Gondar, Gondar, Ethiopia; 3 Vanderbilt University Medical Center, Nashville, TN 37232 USA

**Keywords:** Vaccination, Immunization, Text message, mHealth

## Abstract

**Background:**

The World Health Organization estimates that 29% of under-five mortality could be prevented with existing vaccines. However, non-consistent attendance for immunization appointments remains a global challenge to healthcare providers. Thus, innovative strategies are required to reach the last mile where technology could be effectively utilized to achieve better compliance with children immunization schedules. Therefore, the aim of the review was to systematically collect and summarize the available evidence on the effectiveness of text message reminders on childhood vaccination.

**Methods:**

This review was conducted according to a priori published protocol on PROSPERO. A systematic literature search of databases (PubMed/MEDLINE, EMBASE, Cochrane/Wiley library, and Science direct) was conducted. Eligibility and risk of bias assessments were performed independently by two reviewers. PRISMA flow diagrams were used to summarize the study selection process. Taking into account the level of heterogeneity, a random effects model was used and risk ratios with their 95% CI were used to present the pooled estimates. To investigate the sources of heterogeneity, subgroup analysis and meta-regression analysis were also considered. In this review, publication bias was assessed statistically using Harbord test.

**Results:**

A total of 1771 articles were searched. Out of those 1771 articles, 558 duplicated articles were removed. About 1213 articles were further screened, and finally, ten articles met the inclusion criteria. The meta-analysis showed that there is a significant effect of text message reminders on childhood vaccination coverage (RR = 1.11; 95% CI 1.05–1.17) with a moderate level of heterogeneity (*I*^2^ = 64.3%, *P* = 0.003). The results from the Harbord test suggested that there is no evidence for publication bias (*P* = 0.340).

**Conclusion:**

This review highlights the potential benefits of incorporating mobile text message reminders into the standard management of childhood immunizations, especially in low- and middle-income countries. The frequency and timing of the text message reminders are also crucial in determining the effectiveness of text message reminders. Hence, mHealth interventions deserve more attention as a potential innovation to improve healthcare programs.

**Systematic review registration:**

PROSPERO CRD42017074230

## Background

Vaccinations are widely regarded as one of the most cost-effective public health interventions that help to reduce global child morbidity and mortality. The World Health Organization (WHO) estimates that 29% of under-five mortality could be prevented with existing vaccines [1]. However, vaccine-preventable diseases are still a major cause of morbidity and mortality worldwide, especially in low- and middle-income countries (LMICs) [2].

In 2016, global vaccination coverage has stalled at 86% with an estimated 19.5 million infants worldwide not reached with routine immunization services. As a consequence of this continued failure to reach optimal immunization coverage, 1.5 million children die each year from vaccine-preventable diseases [1].

This high prevalence of childhood vaccine-preventable diseases can be significantly reduced through adherence to confirmed vaccination schedules. However, many barriers to vaccination compliance exist and the proportion of children who had not completed the vaccination program ranged from 23.3 to 76.3% in developing countries [3–5]. The main parental barriers to vaccination include confusion and difficulty in tracking vaccination schedules, low parent knowledge about the benefits of vaccination, missing due dates, and fear of vaccinations’ complications [6–8].

A study in India found the reasons for infants missing vaccination are due to prior reminders not given (32.9%) and parent’s forgetfulness (26.6%) [6] indicating that compliance to the recommended vaccination schedule is a challenge for healthcare systems. Thus, innovative strategies are urgently required where technology could be effectively utilized to achieve better compliance with children immunization schedules.

The increased penetration of Information and Communications Technology (ICT) in health care offers an opportunity to strengthen the health systems [9, 10]. Among the different ICT interventions, mobile phone-based (mHealth) text messaging has gained popularity and may be the key to penetrating hard to reach populations. Text message reminders provide a cost-effective method of relaying health information and reminders [11, 12]. Evidence also showed that these demand-side interventions [13, 14] target characteristics such as forgetfulness in reducing dropouts from vaccination [15–17].

## Justification for the review

Globally, non-attendance for immunization appointments remains a challenge to healthcare providers. In many countries, immunization coverage is low [1], routine immunization systems are weak, and the awareness of the community about the immunization program is low [2, 18]. Though different strategies have been tried to increase the number of children vaccinated, there is a continued failure to achieve vaccination targets [1, 10].

Making well-informed decisions about how best to achieve and sustain high and equitable immunization coverage will depend partly on decision makers accessing the best scientific evidence about what interventions work and integrating this evidence into existing health systems. For any intervention to be adopted in a setting, it must be designed to meet the particular needs of the setting and in the magnitude that best addresses the needs. Although mHealth initiatives are found to be important in health care, its effectiveness varies with implementation context. Therefore, the increased drive to develop and scale-up mHealth interventions with the dynamic ICT environment demands the availability of robust and current evidence.

We set out to explore the evidence around the use of text messaging to improve childhood vaccination, and to our knowledge, found no published systematic review and meta-analysis so far on this topic to help guide decision-making. Therefore, this review would summarize the available evidence on the effectiveness of text message reminders as compared to standard care on childhood vaccination for further public health interventions.

## Methods

### Protocol and registration

This review was conducted according to an a priori published protocol on PROSPERO International Prospective Register of systematic reviews for publication registration number CRD42017074230 (available at https://www.crd.york.ac.uk/PROSPERO/#myprospero).

### Eligibility criteria

PICOS approach was used to set inclusion and exclusion criteria.

#### Inclusion criteria

1. Type of studies: randomized controlled trial studies
2. Types of participants: caregivers/parents of children who are under 5 years of age
3. Types of interventions: we include interventions in which mobile phone text messages provide reminders related to vaccinations
4. Types of controls: studies with comparison group of routine/standard care
5. Types of outcome measures: studies will be eligible for inclusion if they reported vaccination coverages for one of the vaccines or all

#### Exclusion criteria

Studies with no accessible full text, not in English language, and studies which do not report specific vaccination outcomes quantitatively

### Information sources and search strategy

Initially, databases were searched for the same systematic review done before to avoid duplications of efforts. Search terms were pre-defined for a comprehensive search strategy that included text fields within records, and Medical Subject Headings (MeSH terms) were used to expand the searching. We used Boolean operators for the search strategies. A systematic literature search of databases (PubMed/MEDLINE, EMBASE, Cochrane/Wiley library, and Science direct) was conducted. Gray literatures were searched using Google and Google Scholar. In addition, reference lists of relevant studies were identified and reviewed for inclusion (Table 1).

**Table 1:** Search strategy for PubMed

(((((((Child*[MeSH Terms]) OR Child* [All Fields]) OR Infant [MeSH Terms]) OR Infant [All fields])) AND	
(((((((Text messag* [MeSH Terms]) OR Text messag* [All Fields]) OR Telemedicine [MeSH Terms]) OR mhealth [All fields]) OR Reminder systems [MeSH Terms]) OR Reminder systems [All Fields]) OR Messag* [All Fields])) AND	
(((((((((Immuni* [MeSH Terms]) OR Immuni* [All Fields]) OR vaccin* [MeSH Terms]) OR vaccin* [All Fields]) OR “Mass Vaccination” [Mesh Terms]) OR “Immunization Programs” [Mesh Terms]) OR “Immunization Schedule” [Mesh Terms]) OR “Immunization, Secondary” [Mesh Terms]) OR “Immunization, Passive” [Mesh Terms]))	

### Study selection and data collection

Database search results were combined, and duplicate articles were removed using Endnote (version7) and manually. Eligibility assessment was performed independently by two reviewers (Zeleke Abebaw and Kasahun Dessie). Discrepancies between the two reviewers in this process were discussed with the other review team member (Binyam Tilahun) until consensus was reached. Articles identified through the electronic literature searches and from other sources were comprehensively reviewed based on the eligibility criteria for inclusion. The methods for data collection and analysis were based on the Cochrane Handbook of Systematic Reviews for Interventions [19].

### Quality assessment (risk of bias) in individual studies

Two authors (Zeleke Abebaw and Kashaun Dessie) independently assessed the risk of bias for each included study by using the Cochrane Collaboration Risk of Bias Tool [19]. Articles were scored as having low, high, or unclear risk of bias for the seven attributes: sequence allocation, allocation concealment, blinding of participant and personnel, blinding of outcome assessor, incomplete outcome data, selective reporting, and other sources of bias. The two authors resolved disagreements in the assessment of risk of bias by discussion and consensus, consulting a third author (Binyam Tilahun) for any persistent disagreements. The kappa statistic was used to assess the level of agreement during risk of bias assessment by the two authors.

### Data extraction and management

Data were extracted by two authors (Zeleke Abebaw and Kasahun Dessie) using a standardized data extraction format. The two authors’ resolved disagreements by discussion consulting a third author (Binyam Tilahun) for any persistent disagreements. The final data were entered into the Cochrane Collaboration Review Manager Version 5.3 statistical software for risk of bias analysis. Finally, Stata version 14 software was used to conduct the meta-analysis.

### Assessment of heterogeneity

Heterogeneity in meta-analysis refers to the variation in study outcomes between studies where *I*^2^ statistic describes the percentage of variation across studies that is due to heterogeneity rather than chance [20]. We quantified the level of statistical heterogeneity with *I*^2^ of 50% or more indicating the presence of substantial heterogeneity which is an indication to use random effects model and to perform subgroup analysis or meta-regression [20]. We reviewed clinical heterogeneity in the setting, participants, intervention, and outcomes of included studies in order to make a qualitative assessment of the extent to which the included studies were similar to each other. Forest plot was used visually to assess the level of heterogeneity. In addition, Cochrane’s *Q* statistic was performed with a *P* value for the chi-squared test of less than 0.1 indicating the presence of statistical heterogeneity.

### Assessment of publication bias

Publication bias occurs when the results of published studies are systematically different from the results of unpublished studies. In this review, evidence of publication bias was investigated by examining the symmetry of the funnel plot and by performing a Harbord statistical test [20].

### Measures of effect and reporting

Systematic reviews and meta-analyses are essential tools for summarizing evidence for which PRISMA statement flow diagrams ensure transparent and complete reporting [21]. Forest plots and funnel plots were used to visualize the level of heterogeneity and publication bias, respectively. Random effects model was used during analysis, and risk ratios with their 95% CI were used to present the pooled effect sizes [20].

### Assessment of certainty of evidence

We assessed the certainty of evidence using GRADE (Grading of Recommendations, Assessment, Development and Evaluation). This method results in an assessment of the quality of the body of evidence as high, moderate, low, or very low. Body of the evidence was assessed against the following criteria: risk of bias, heterogeneity, imprecision, indirectness, and publication bias [19].

## Results

### Selection of studies

**Fig. 1:**
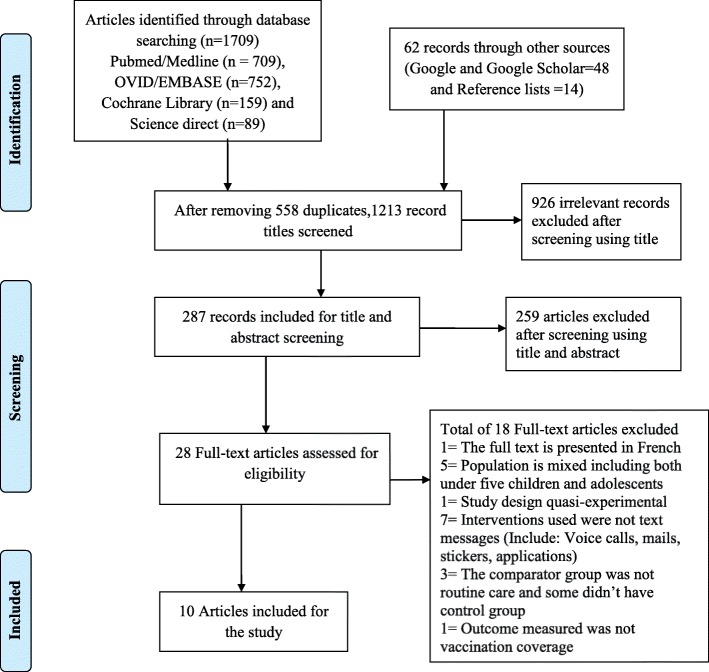
PRISMA flow diagram representing the study selection process [21]

A total of 1771 articles were searched. Out of those 1771 articles, 558 duplicated articles were removed using Endnote7 software. Finally, the 1213 articles were screened based on the eligibility criteria. After screening of titles and abstracts, 28 articles were included for full-text screening based on the eligibility criteria (Fig. 1). Finally, 10 studies were eligible and included for the review [22–31], while the remaining 18 full-text articles were excluded with reasons (Table 3 in the Appendix).

### Characteristics of included studies

A total of ten articles met the inclusion criteria. These studies were conducted in the USA (5), Kenya (2), Nigeria (1), Guatemala (1), and Zimbabwe (1) (Table 2). Author, year of publication, country, study design, study setting, population, intervention used, duration of intervention, timing of message, vaccine type, and outcome data were extracted to describe the characteristics of the studies. The total sample size of the studies was 10,625 (5273 in the intervention group and 5325 in the control groups). Majority (60%) of the studies enrolled parents/women from health facilities [22, 23, 26, 29–31], three enrolled children who presented for a first dose [24, 25, 28], and one study enrolled caregivers at the village level [27].

**Table 2:** Characteristics of included studies

S. no	Author (year)	Country	Study design	Study participants/setting	Population (I, C)	Interventions/control	Follow-up periods	Outcome measure	Event among intervention	Event among control
1	Bangure et al. (2013)	Zimbabwe	RCT	Women who delivered were recruited into the study at 4 Kadoma city clinics in Mashonal and West province of Zimbabwe	304 (152, 152)	SMS reminders were sent at 6, 10, and 14 weeks on 7, 3, and 1 day before vaccination due dates. Control group received routine care.	14 weeks	Immunization coverage at 6, 10, and 14 weeks	144/152	114/152
2	Schmidt et al. (2012)	USA	RCT	Parents of newborns being discharged from a local hospital who intended to seek child health care at the university-sponsored pediatric resident and faculty clinic	68 (28, 40)	A text message reminder sent 7 days prior to vaccination schedules. Control group received standard notifications.	7 months	Receipt of vaccinations due at 2, 4, 6 months of age	22/28	31/40
3	Dini et al. (2000)	USA	RCT	Households in the Denver metropolitan area whose children had received the first dose of DTP and/or poliovirus vaccines	960 (440, 520)	Reminder messages sent on due dates of vaccination. Control group has no notifications.	24 months	Vaccination series completion at 24 months of age	217/440	213/520
4	Haji et al. (2014)	Kenya	RCT	Children < 12 months of age who were brought to the selected vaccinating health facilities in the three districts of Machakos, Langata and Njoro for their first dose of pentavalent vaccine	744 (372, 372)	Text reminders sent 2 days before and on the day of the scheduled Vaccination due date. Control group received routine reminder.	14 weeks	Receipt of scheduled vaccines at 10 and 14 weeks	359/372	309/372
5	Eze GU et al. (2015)	Nigeria	Parallel group RCT	Paired child-caregivers were followed-up at 8 health facilities in an urban/sub-urban area in South Nigeria	905 (452, 453)	A text message reminder sent a day before the due date. Control group received routine care.	18 weeks	DPT3 Coverage at 18 weeks	312/452	273/453
6	Niederhauser et al. (2015)	USA	RCT	The study enrolled parent/infant dyads recruited from a large federally qualified health center, a Women, Infants and Children clinic, a private pediatric clinic and at the Honolulu Baby Expo	42 (19, 23)	Text message sent 4 weeks and 2 weeks prior to the due date for the infant’s 2, 4, and 6-month vaccinations. Control group received routine care.	7 months	5 vaccines (DTaP, HepB, HIB, PCV, and polio) complete coverage at specific ages by group	7/19	15/23
7	Domek et al. (2016)	Guatemala	Pilot RCT	Infants aged 8–14 weeks presenting for the first dose of the primary immunization series at two public health clinics were enrolled	321 (160, 161)	SMS text messages at 6, 4, and 2 days before the next scheduled date for visits 2 and 3.	7 months	Completing all (pentavalent, pneumococcal, polio, and rotavirus) vaccines	130/160	122/161
8	Gibson et al. (2017)	Kenya	Clustered RCT	Villages were randomly and evenly allocated. Caregivers were eligible if they had a child younger than 5 weeks who had not yet received a first dose of pentavalent vaccine	748 (388, 360)	SMS reminders sent 3 days and the day before vaccination visits at 6, 10, and 14 weeks and at 9 months. Control group with no SMS service.	12 months	Full coverage (BCG, three doses of polio, three doses of pentavalent and measles)	332/388	296/360
9	Hofstetter et al. (2015)	USA	RCT	Parents of 9.5–10.5-month-old children from four urban academically affiliated pediatric clinics	1368 (686, 682)	Text reminder sent 2 days before the schedule. Control arm with usual care.	13 months	MMR vaccination by 13 months	263/686	270/682
10	Stockwell et al. (2012)	USA	RCT	Four community-based clinics in the USA	5165 (2576, 2589)	Text message reminders sent a month before due date. Control received usual care.	12 months	Receipt of an influenza vaccine	1316/2576	1202/2589

*I* intervention, *C* control

Of the included studies, five (50%) were published after 2015. The studies were mostly conducted in the USA, 5 (50%), followed by Kenya, 2 (20%). The follow-up period between studies varied from 14 weeks to 2 years with very high lost to follow-up in two studies [22, 30]. The frequency of messages and timing of the text message reminders was different in the included studies where two studies [23, 25] sent the reminders three times for a single schedule. Four studies [24, 25, 27, 30] measured full vaccination coverage, while three studies measured the outcome for three doses of coverage [22, 23, 26]. The remaining studies measured the outcome at two doses [28] or one dose [29, 31] (Table 2).

### Quality (risk of bias) assessment for the included studies

The risk of random sequence generation and allocation concealment was low for 80% of studies while unclear for the remaining two studies [24, 28]. Risk of bias in relation to blinding of participant and personnel was high for two studies [27, 29], while risk of bias in blinding of outcome assessments was low for majority of the studies. The risk of attrition bias was high for three studies [22, 29, 30] and low risk for the remaining studies. The risk of selective reporting was high for one study [29] and low for the remaining studies. The kappa statistic showed that level of agreement during risk of bias assessment by the two authors (Zeleke Abebaw and Kasahun Dessie) was 69.2% (*P* value < 0.0001) (Fig. 3 in the Appendix).

### Quantitative data synthesis

#### Uptake of vaccination in all included studies

**Fig. 2:**
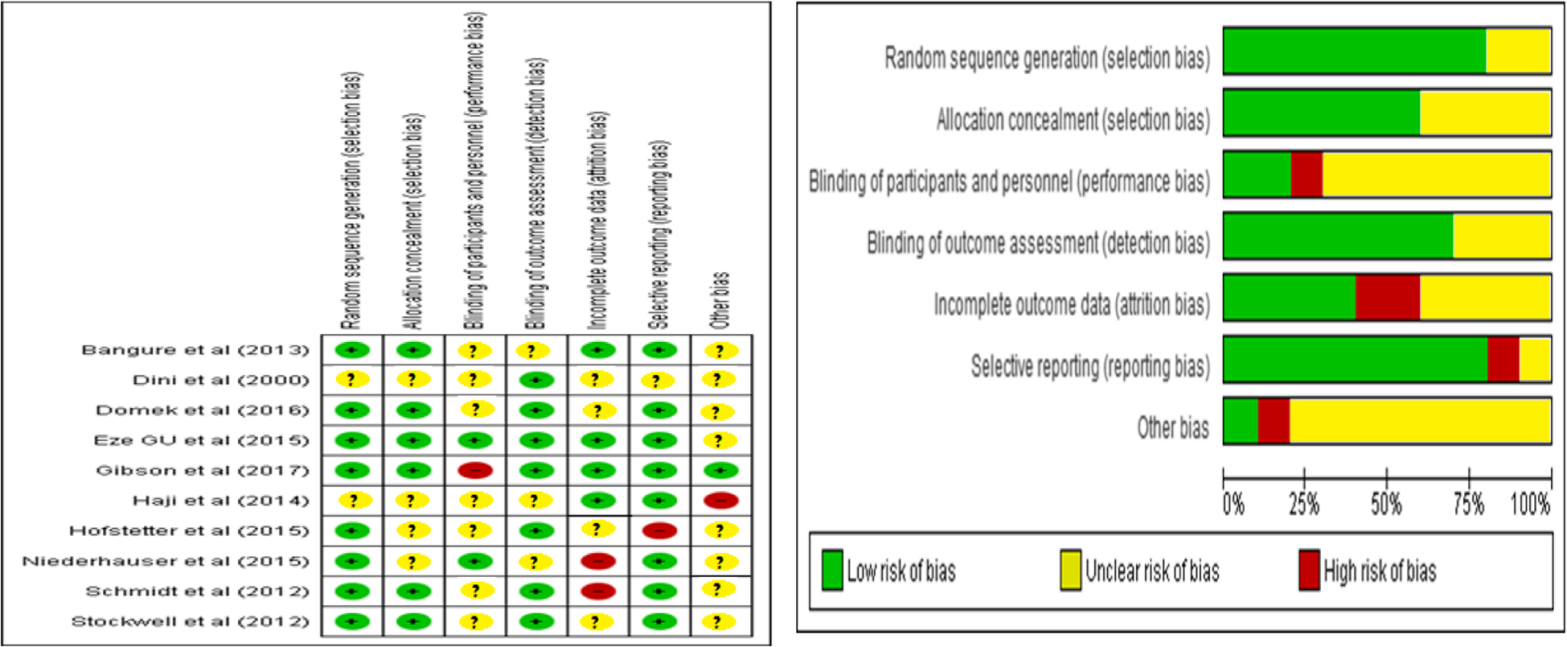
Forest plot showing the pooled estimate and level of heterogeneity

**Fig. 3:**
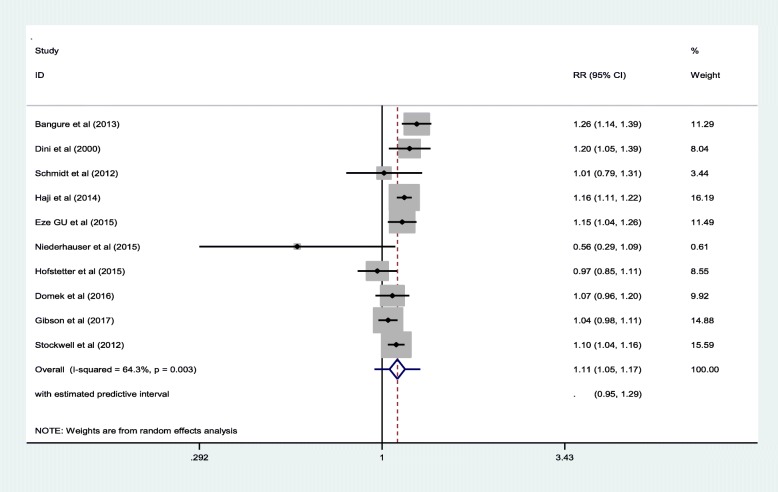
Risk of bias assessment for the included studies

The meta-analysis findings showed that there is a moderate level of statistically significant heterogeneity (*I*^2^ = 64.3%; *P* = 0.003) (Fig. 2). Since the *I*^2^ (64.3%) was greater than 50%, random effects model was used to estimate the pooled estimates. Accordingly, the meta-analysis revealed that there is a significant effect of text message reminders on childhood vaccination coverage (RR = 1.11; 95% CI 1.05–1.17).

The predictive interval in this review overlaps the null (0.95, 1.29), indicating considerable uncertainty about the size and direction of an effect in a new study (Fig. 2).

### Uptake of vaccination by subgroups

The magnitude of the heterogeneity (64.3%) also showed that there is a need to conduct subgroup analysis to investigate the sources of heterogeneity.

### Subgroup analysis results by country income status

In studies from high-income countries, the effect of a text message on childhood vaccination was positive and statistically insignificant (RR = 1.06; 95% CI 0.96–1.18) with moderate heterogeneity (*I*^2^ = 56.6%). The predictive interval in this review overlaps the null (0.78, 1.45), indicating considerable uncertainty about the size and direction of an effect in a new study.

**Fig. 4:**
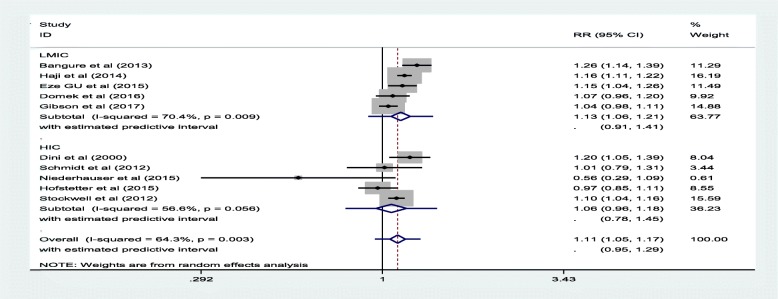
Subgroup analysis by countries’ income status

On the other hand, there was a statistically significant positive effect of text message reminders among LMICs (RR = 1.13; 95% CI 1.06; 1.21) with moderate heterogeneity (*I*^2^ = 70.4%). The predictive interval in this review overlaps the null (0.91, 1.41), indicating considerable uncertainty about the size and direction of an effect in a new study. The test for subgroup differences also indicated that there is no statistically significant difference across subgroups (*P* = 0.31) (Figure 4 in the Appendix).

### Subgroup analysis by timing of last message

**Fig. 5:**
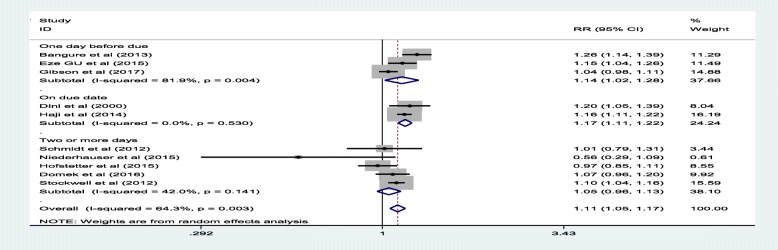
Subgroup analysis by timing of the last text message reminder

The forest plot showed that those studies in which the last reminder messages were sent on the due date had a statistically significant positive effect (RR = 1.17; 95% CI 1.11–1.22) on childhood vaccination with no heterogeneity (*I*^2^ = 0%). Similarly, sending reminder text messages 1 day before the due date also had a significant positive effect (RR = 1.14; 95% CI 1.02–1.28) on childhood immunization with substantial heterogeneity (*I*^2^ = 81.9%). However, sending the text messages 2 or more days before the due date has no significant effect on childhood immunization coverage (RR = 1.05, 95% CI 0.96–1.13) with low heterogeneity (*I*^2^ = 42%). The test for subgroup differences also indicated that there is no statistically significant difference across subgroups (*P* = 0.07) (Figure 5 in the Appendix).

### Subgroup analysis by number of messages for a single schedule

**Fig. 6:**
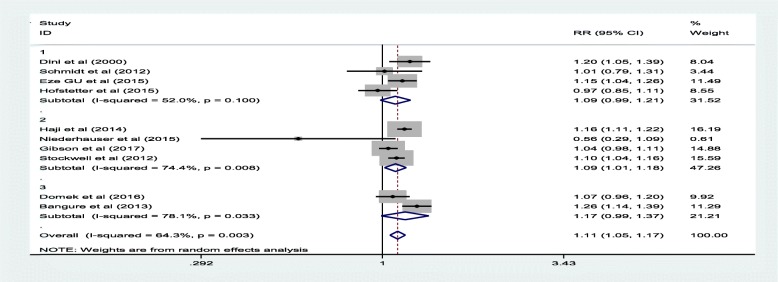
Subgroup analysis by number of messages for a single schedule

The result showed that the number of messages sent for a given vaccination appointment schedule had different effects on childhood coverage. Accordingly, sending two text messages for one schedule visit had shown a statistically significant effect on childhood vaccination (RR = 1.09; 95% CI 1.01–1.18) with moderate heterogeneity (*I*^2^ = 74.4%). The test for subgroup differences also indicated that there is no statistically significant difference across subgroups (*P* = 0.76) (Figure 6 in the Appendix).

### Meta-regression

**Fig. 7:**
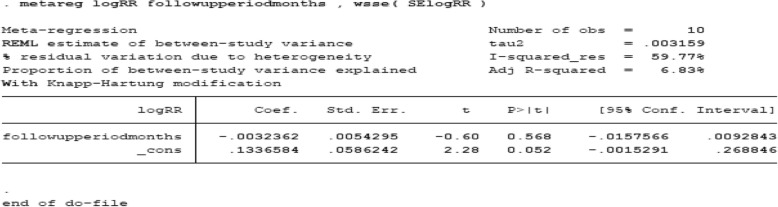
Outputs of meta-regression analysis

Meta-regression is a tool used in meta-analysis to examine the impact of variables on the pooled effect size using regression-based techniques [20]. In this review, the effect of each individual study’s follow-up time on the pooled estimate was assessed using meta-regression. Though not statistically significant, the findings revealed that an increase in a month of follow-up at which outcome was measured corresponds to a decrease in the logRR of intervention effect by 0.003 (*P* value = 0.568) (Figure 7 in the Appendix).

### Assessment of publication bias

**Fig. 8:**
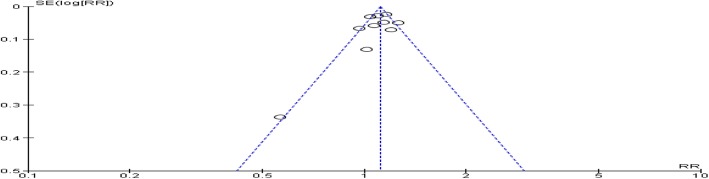
Funnel plot to assess publication bias

**Fig. 9:**
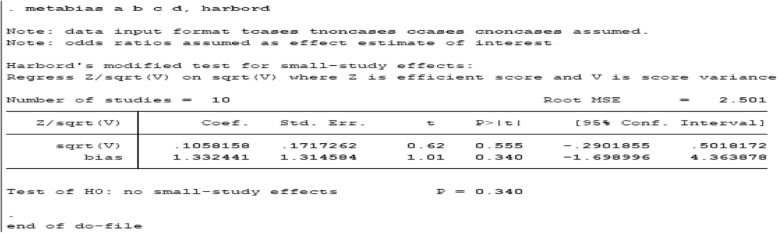
Outputs of the Harbord test to assess publication bias

A funnel plot provides evidence on the presence of small study effects based on subjective visual inspection. If the individual plots are located symmetrically in the funnel plot, it indicates the absence of publication bias [20]. In this review, the funnel plot observation suggests the absence of publication bias (Figure 8 in the Appendix). To objectively measure the presence of publication bias, a Harbord test was also conducted. The results from the Harbord test suggested that there is no evidence for publication bias (*P* value, 0.340) (Figure 9 in the Appendix).

### Certainty of evidence

**Fig. 10:**
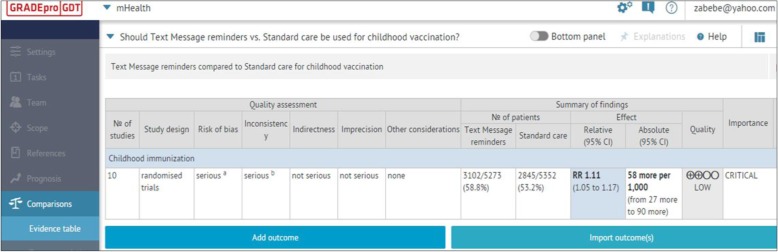
Assessment of certainty of evidence using GRADEpro software

The GRADE approach was used to assess the quality of evidence for systematic review and meta-analysis based on defined parameters as high, moderate, low, or very low [19]. In this review, the risk of bias was serious and there was moderate heterogeneity among studies making the quality of the body of evidence low level (Figure 10 in the Appendix).

## Discussion

Multiple established and emerging strategies have been implemented to foster vaccination coverage globally. Despite the vast investment of resources in improving vaccination coverage in LMICs, few studies are available to inform policy and decision-making on childhood vaccination. In this review, ten articles were included to estimate the best available evidence on the effects of text message reminders in increasing vaccination coverage among children less than 5 years of age.

The pooled estimate revealed that there is a potential for text message reminders to improve childhood vaccination rates. However, the included studies showed moderate heterogeneity, which could result from clinical differences across studies. The precise effectiveness of these interventions is likely to be influenced by numerous factors such as country setting and nature of the interventions used. We did not find any important changes in the level of heterogeneity when we investigated the effect by country setting and number of text messages sent. However, the level of heterogeneity became lower in studies with text messages sent on the due date of vaccination with statistically significant positive effect.

Sending the last text message reminder nearer to the vaccination appointment date was also found to be more important since tackling forgetfulness needs more recent information with immediate actions.

We found relatively lower effectiveness of text reminders in high-income countries, despite the much greater level of economic advancement and immunization infrastructure in these areas than low- and middle-income countries. Differences in study settings could explain some of the observed differences. For example, studies done in the USA were more likely to include participants who presented to healthcare facilities and thus had enough resources for healthcare access (facility-based studies).

We used the GRADE approach to assess the level of confidence to be placed on the evidence for the effects of text message reminders on vaccination coverage [19]. Randomized trials without important limitations provide high-quality evidence. However, in three of the included studies, the risk of attrition bias was high and in two studies the risk of blinding of personnel and participant was also high (Figure 10 in the Appendix). This led us to downgrade the quality of the evidence from high to moderate [19]. In addition, the presence of moderate heterogeneity further downgrades the quality of the evidence to a low level. Low quality of evidence implies the likely effect of text message reminders on childhood vaccination coverage with low confidence on the estimates to be implemented globally.

The findings from our review are in line with the findings of other reviews done on vaccination uptakes among adolescent and pregnant women where text messages are found to be effective reminders [13, 32, 33]. Stockwell et al. also presented a literature review of a broad range of health information technologies to improve vaccine communication and coverage and suggested that SMS is effective in improving vaccination uptake with the potential to penetrate large populations and relatively low cost [12]. Text messaging was also shown to be effective in non-vaccination-related health conditions [34]. The WHO has also reported that mHealth technologies are promising to strengthen health systems [35].

### Strengths and limitations

The main strength of the current review lies in our adherence to international standardized guidelines on the conduct and reporting of systematic reviews. We included studies only from peer-reviewed English-language journals, which may have restricted our findings.

## Conclusions

Mobile phone text message reminders have a potential to improve childhood vaccination coverages. This review highlights the potential benefits to childhood vaccine uptake of incorporating text message reminders into the standard management of childhood immunizations especially in low- and middle-income countries. The frequency and timing of the text message reminders are crucial in determining the effectiveness of text message reminders on childhood vaccination.

### Implications for practice

The evidence presented in this review shows promise for the use of text message reminders to strengthen immunization programs. Thus, text message reminders deserve more attention as a potential innovation to improve healthcare operations more particularly in developing countries, where the mobile phone penetration is growing exponentially.

### Implications for future research

Current evidence around mHealth interventions to improve vaccination is of low quality and with few studies. Therefore, more research is needed in this area.

## Data Availability

The extracted data is available upon request.

## Appendix

**Table 3:** Excluded studies with reasons

S. no.	Authors (year)	Reasons for exclusion
1	Li Chen (2016)	The intervention was EPI application and text reminder sent for both groups
2	Wakadha et al. (2013)	It was a feasibility study with no comparison group of standard care
3	Uddin et al. (2016)	It was quasi-experimental study where the outcome was measured pre and post intervention in both groups
4	Schlumberger et al. (2015)	The abstract was in English but full document was in French. Though the author was contacted but unable to get the English version of the full text
5	Abramson et al. (1995)	Interventions were reminder cards and phone calls
6	Morgan et al. (1998)	Interventions were telephone call and mailed reminders
7	Aragones et al. (2015)	Population includes children above 5 years of age
8	Bjornson et al. (1999)	Interventions were mailed reminders
9	Kempe et al. (2015)	It was a pragmatic trial with interventions mail and autodialed telephone calls
10	Stockwell et al. (2015)	Population extends above 5 years (6 months up to 8 years) with no disaggregation for under-five children
11	Zhou-Chen et al. (2008)	Outcome was improving attendance rates at a health promotion center
12	Macknin et al. (2000)	Population extends beyond under 5 years (0–18 years) and no separate data for children
13	Sther–Geen et al. (1993)	Interventions were voice calls
14	Hofstetter et al. (2015)	Population beyond under 5 years (6 months to 17 years) with educational and interactive messages
15	Lieu et al. (1998)	The study did not randomize patients to no intervention group
16	Dombkowski et al. (2014)	Intervention was mailed recall and reminder
17	O’Leary et al. (2015)	Population was adolescents without under-five children
18	Irigoyen et al. (2000)	Interventions were phone calls and post card reminders

## Abbreviations

CI: Confidence interval
GRADE: Grading of Recommendations, Assessment, Development and Evaluation
MeSH: Medical Subject Headings
PRISMA: Preferred Reporting Items for Systematic Reviews and Meta-Analysis
RCT: Randomized controlled trial
RR: Relative risk
SRMA: Systematic review and meta-analysis
WHO: World Health Organization
